# Regulation of Tumor Immunity by Tumor/Dendritic Cell Fusions

**DOI:** 10.1155/2010/516768

**Published:** 2010-10-26

**Authors:** Shigeo Koido, Sadamu Homma, Eiichi Hara, Yoshihisa Namiki, Akitaka Takahara, Hideo Komita, Eijiro Nagasaki, Masaki Ito, Toshifumi Ohkusa, Jianlin Gong, Hisao Tajiri

**Affiliations:** ^1^Division of Gastroenterology and Hepatology, Department of Internal Medicine, The Jikei University School of Medicine, Tokyo 277-8567, Japan; ^2^Institute of Clinical Medicine and Research, The Jikei University School of Medicine, Tokyo 277-8567, Japan; ^3^Department of Oncology, Institute of DNA Medicine, The Jikei University School of Medicine, Tokyo 105-8461, Japan; ^4^Saitama Cancer Center Research Institute for Clinical Oncology, Saitama 362-0806, Japan; ^5^Department of Medicine, Boston University School of Medicine, Boston, MA 02118, USA

## Abstract

The goal of cancer vaccines is to induce antitumor immunity that ultimately will reduce tumor burden in tumor environment. Several strategies involving dendritic cells- (DCs)- based vaccine incorporating different tumor-associated antigens to induce antitumor immune responses against tumors have been tested in clinical trials worldwide. Although DCs-based vaccine such as fusions of whole tumor cells and DCs has been proven to be clinically safe and is efficient to enhance antitumor immune responses for inducing effective immune response and for breaking T-cell tolerance to tumor-associated antigens (TAAs), only a limited success has occurred in clinical trials. This paper reviews tumor immune escape and current strategies employed in the field of tumor/DC fusions vaccine aimed at enhancing activation of TAAs-specific cytotoxic T cells in tumor microenvironment.

## 1. T Lymphocytes and Tumor Immunity

**Figure 1:**
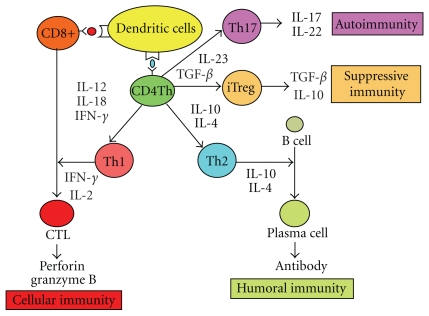
The role of helper T cells in tumor immunity. CD4+ T-helper cells play extensive roles and are able to interact with the tumor cell and immune effectors. Th1 cells secrete type I cytokines such as interleukin 2 (IL-2) and IFN-*γ*, resulting in the activation of DCs, which can stimulate CTLs. Tumor-specific Th1 cells regulate the survival and persistence of CD8+ effector T cells as memory cells. Th2 cells secrete type II cytokines, such as IL-4 and IL-10. Th2 cells can enhance the generation of humoral, antibody-based antitumor responses. Th17 cells secrete IL-17 elicit tissue inflammation implicated in autoimmunity. Inducible CD4+ regulatory T cells (iTreg) exhibit a strong immunosuppressive activity for antitumor immunity.

The T-cell receptor (TCR) interaction with complex of peptides and major histocompatibility complex (MHC) molecules is a critical event in T-cell-mediated responses. The proteasomes in tumor cells degrade tumor-associated antigens (TAAs) into short peptides (usually 8–10 amino acids), mostly derived from endogenously synthesized proteins as well as exogenous antigens in the endoplasmic reticulum, and present them to cytotoxic T lymphocytes (CTLs) that express the CD8 coreceptor. Therefore, CD8+ CTLs can directly lyse tumor cells [1, 2]. On the other hand, CD4+ T cells recognize antigenic peptides (10–30 amino acids) associated with MHC class II molecules and mediate their helper functions to induce antigen-specific CTLs through secretion of cytokines such as interferon (IFN)-*γ*. There are increasing evidences that CD4+  T cells play a more direct role beyond delivery of assistance in the generation of efficient stimulatory immunity [3]. CD4+ T-cell responses can also elicit not only stimulatory but also suppressive immunity. Now, it is becoming clear that there is an enormous diversity in CD4+ T-helper (Th) cell polarization patterns including Th1, Th2, Th17, and regulatory T cells (Tregs). Th1 cells secrete type I cytokines such as IFN-*γ*, resulting in the activation of antigen presenting cells (APCs), which can stimulate CTLs [1, 2]. Tumor-specific CD4+ T cells regulate the survival and persistence of CTLs as memory cells [3]. Both CD8+ and Th1 cells secrete IFN-*γ*, which can further sensitize tumor cells to CTLs by upregulating MHC class I molecules and antigen-processing machinery of APCs. Th2 cells secrete type II cytokines, such as interleukin 4 (IL-4) and IL-10 [1, 2]. Th2 cells can enhance the generation of a humoral immunity, antibody-based antitumor response. The newly identified Th17 cells secrete IL-17, eliciting tissue inflammation implicated in autoimmunity. Finally, Tregs inhibit the development of CTL responses [4]. Tregs are mainly derived from two origins, which are naturally occurring thymus-derived Tregs (nTregs) and adaptive or inducible Tregs (iTregs) [5]. Foxp3 has been considered to be a master regulatory transcription factor for Tregs [6]. It is becoming clear that Tregs play a pivotal role in the tumor progression and the suppression of tumor immunity [7] (Figure 1).

## 2. Dendritic Cells (DCs) and Tumor Immunity

 Dendritic cells (DCs) are professional APCs and key regulators of  T- and B-cell immunity, owing to their superior ability to take up, process, and present TAAs [1, 2, 8]. DCs derive their potency from constitutive and inducible expression of essential costimulatory ligands on the cell surface including B7, ICAM-1, LFA-1, LFA-3, and CD40 [9, 10]. These proteins function in concert to generate a network of secondary signals essential for reinforcing the primary antigen-specific signal in T-cell activation [11, 12]. Therefore, DCs play a pivotal role on the initiation, programming, and regulation of tumor-specific immune responses. Various strategies to deliver TAAs into DCs have been developed to generate potent CTL responses against tumor cells. DCs have been pulsed with synthetic peptides derived from the known TAAs, tumor cell lysates, apoptotic tumor cells, and tumor RNA [13–17]. Another strategy is the use of fusion cells generated by fusing DCs and whole tumor cells [18]. The fusion process facilitates the entry of TAAs, including both known and unidentified, into the endogenous antigen-processing pathway and presents antigenic peptides through MHC class I and II pathways in the context of the potent immune-stimulatory machineries in the DCs [19–22]. These antigen-loaded DCs have already been used as vaccines to improve antitumor immunity [8].

## 3. Fusions of Tumor Cell and DC

**Figure 2:**
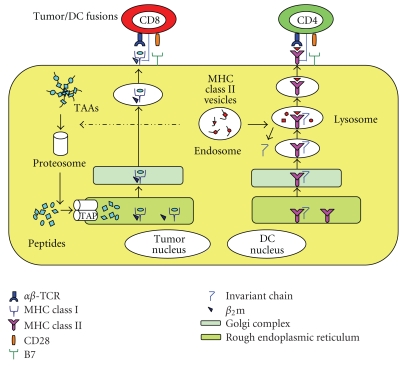
Characterization of tumor/DC fusions. Tumor/DC fusions express MHC class I, II, costimulatory molecules and tumor-associated antigens (TAAs). The fusions are able to process tumor-derived peptides and MHC class I peptides derived from DCs. They form MHC class I-peptide complexes, in the endoplasmic reticulum, which are transported to the cell surface and presented to CD8+ T cells. Similarly, the fusions can synthesize MHC class II peptides derived from DC in the endoplasmic reticulum, which are transported to the cytoplasm where MHC class II-peptide complexes are assembled with tumor-derived peptides and presented to CD4+ T cells.

The fusions with whole tumor cell and DC (tumor/DC) by polyethylene glycol (PEG) known as a chemical membrane destabilizing agent [18, 23–25], physical [26–31], or biological means [32, 33] create heterokaryons that express both TAAs and DC-derived costimulatory molecules. Therefore, the fused cells inherit the properties of their parental cells (tumor cell and DC) (Figure 2). For example, the membranes of fused cells are integrated into a single cell whereas the nuclei are remained to be separate, at least in the primary fusions [34]. Such a characteristic structure may make it possible to maintain the functions of both original cells, at least in part, including synthesis of antigens and costimulatory molecules [34].

## 4. Antigen Processing and Presentation by Tumor/DC Fusions

It has been shown that antigens are processed and presented through two major pathways by DCs. Endogenously synthesized proteins, such as those expressed in viral infections and certain exogenous antigens are processed and presented through the MHC class I-restricted pathway to CD8+ T cells [35, 36]. In contrast, exogenous antigens from the extracellular environment are captured and delivered to the compartments of the endosome/lysosome, where they are degraded to antigenic peptides by proteases and peptidases, which are complexed with MHC class II molecules and recognized by CD4+  T cells [35, 36]. Importantly, DCs are also capable of processing and presenting exogenous antigens on MHC class I molecules through an endogenous pathway, a phenomenon called antigen cross-presentation [37, 38]. However, the antigen cross-presentation is generally not efficient to induce CTL responses in the absence of carrier proteins or particles [39]. 

It is now well known how the fusion cells assemble and present the MHC class I- and II-restricted peptide complexes. One possibility is that antigenic peptides are complexed with tumor-derived MHC class I molecules and the complexes are simply transferred and presented by tumor/DC fusions. Moreover, the fusions can efficiently process TAAs from tumors through an endogenous antigen-processing pathway [34]. Therefore, an advantage of the fusions-strategy over DCs pulsed with tumor lysates is that endogenously synthesized antigens have better access to MHC class I pathway [40]. Indeed, tumor/DC fusion vaccines are superior to those involving other methods of DCs loaded with antigenic proteins, peptides, tumor cell lysates, or irradiated tumor cells in animal studies [41]. Moreover, the important advantage of tumor/DC fusions approach is that modifications of tumor cells and DCs are independently possible, which their characters persist after fusion process [22].

## 5. CTL Induction by Tumor/DC Fusions

Immature DCs take up tumor antigens, mature into IL-12-producing cells, and stimulate Th1 cells in the draining lymph node, resulting in IFN-*γ* production. These stimulated Th1 cells help during the priming of CD8+  T cells with the capacity for optimal secondary expansion upon re-encounter with antigens. Even in the absence of CD4+ T cells, these memory CD8+ T cells can be rapidly expanded in response to secondary antigens exposure. Expanded CD8+ CTLs can destroy tumor cells through effector molecules such as granzyme B and perforin [42]. Therefore, efficient CTL induction requires the stimulation of both CD4+ and CD8+ T cells. Expression of MHC class I and II molecules, costimulatory molecules (CD80 and CD86), and adhesion molecules (ICAM-1 and LFA-3) on tumor/DC fusions is essential for antigen processing, presentation, and subsequent activation of both CD4+ and CD8+ T cells [25, 43, 44]. In animal models, the fusion cells, like DCs, can also migrate into regional lymph node as early as 18 hours after s.c. injection. Then, the fusion cells localize to the T-cell area in the lymph node and form clusters with CD4+ and CD8+ T cells simultaneously [45].

To dissect the role of antigen-presentation through MHC class I and II pathways by tumor/DC fusions, we created four types of fusions by alternating fusion cell partners: (1) wild-type fusions (WT-FCs), (2) MHC class I knockout fusions (IKO-FCs), (3) MHC class II knockout fusions (IIKO-FCs), and (4) MHC class I and II knockout fusions (I/IIKO-FCs) [46]. Immunization of wild-type mice with WT-FCs, IKO-FCs, IIKO-FCs, or I/IIKO-FCs provided 100, 91.7, 61.5, and 15.4% protection, respectively, against tumor challenge with MHC class I positive tumor cells. Moreover, IKO-FCs induced slightly decreased tumor prevention and treatment. Importantly, IIKO-FCs abolished IFN-**γ**production of CD4+ and CD8+ T cells and CTLs induction. Therefore, antigen presentation through MHC class II is essential for the activation of antigen-specific CD4+ T cells and the induction of potent CD8+ CTL responses against tumor. Although development of vaccine has been directed toward activation and amplification of CD8+ T cells, there is increasing evidence that CD4+ T cells play a broader role in antitumor immunity [3]. CD4+ T cells contribute to antitumor immunity through diverse mechanisms, in which they are required not only for the maintenance of CD8+ CTLs but also for the infiltration of CD8+ CTLs at the tumor site [3]. Indeed, adoptive transfer of antigen-specific CD4+ T cells controlled tumor growth [46]. Although maximal antitumor immune responses require both MHC class I and II antigen-presentation, MHC class II plays more important roles on the antitumor immunity in cancer vaccines [3, 46]. Therefore, for the design of cancer vaccines, it is essential for activating robust and long-lasting CD4+ and CD8+ T cell responses in patients with cancer.

## 6. Tumor/DC Fusions Vaccine

Tumor/DC fusions have been strongly effective in animal studies using melanoma [26, 31, 31, 47–52], colorectal [18, 30, 45, 50, 51, 53–59], breast [60–65], esophageal [66], pancreatic [67, 68], hepatocellular [69–73], lung [74–78], renal cell carcinoma [79], sarcoma [80–85], myeloma [86–93], mastocytoma [94], lymphoma [95], and neuroblastoma [96]. More importantly, in preclinical studies the fusions were also effective to induce CTL responses in vitro using colorectal [25, 97–102], gastric [103, 104], pancreatic [105], breast [43, 106–110], laryngeal [111], ovarian [34, 44, 112], lung [113], prostate [114, 115], renal [116, 117], and hepatocellular [118–120] carcinoma, leukemia [121–126], myeloma [127, 128] sarcoma [129, 130], melanoma [29, 131–133], glioma [124], and plasmacytoma [134]. 

Based on these unique features of tumor/DC fusions with antitumor immunity in murine and preclinical studies, initial Phase I/II clinical trials have been conducted in a variety of tumors (Table 1). Tumor/DC fusions vaccine was first reported in patients with melanoma. Allogeneic DCs were fused with autologous melanoma cells by electrofusion and vaccinated in 16 patients with disseminated melanoma refractory to standard therapy [135, 136]. There were no serious side effects associated with the administration of the vaccine. Seven of the 16 patients responded to the vaccination, one with complete response, one with partial response, and five with stable disease, following to previous rapid progression. Similar results in patients with melanoma were reported from another group using autologous melanoma cells fused to DCs either from healthy donors [137] or the patients [138]. Although Tumor/DC fusions vaccine was also coadministrated with rIL-2, efficient antitumor immunity was not observed in patients with melanoma [139]. Moreover, vaccination with fusions of HLA class I-mismatched DCs from healthy donor and autologous melanoma cells failed to find unequivocal beneficial effects [139]. In addition, in malignant glioma, autologous fusions vaccine produced partial clinical responses in two of six patients [140]. In a similar trial by the same group, a combination of autologous fusions and rIL-12 was administered to patients with malignant glioma, melanoma, breast, gastric, colorectal, and ovarian cancer [23, 24, 141]. Three of 12 patients with malignant glioma achieved a partial response and one patient a minor response [24] but the response to other types of malignant tumors was muted [23]. Another group tested fusions vaccine in 23 patients with metastatic breast and renal cancer [142]. Immunologic and clinical responses were observed in a subset of patients. Two patients with breast cancer exhibited disease regression, including a nearly complete response of a large chest-wall mass. Five patients with renal cell carcinoma and one patient with breast cancer showed stable disease. In a subsequent trial from same group, autologous renal cell carcinoma cells were fused with allogeneic DCs [143]. Although antitumor immune responses were observed in 10/21 evaluable patients, a partial clinical response was demonstrated in two patients and stable disease in eight patients. In patients with renal cell carcinoma, fusions vaccine generated with allogeneic DCs and autologous tumor cells showed immunologic, but not effective clinical responses [138, 144, 145]. Together, only limited therapeutic results were obtained in all these clinical trials.

## 7. Immunosuppression in Tumor Microenvironment

**Figure 3:**
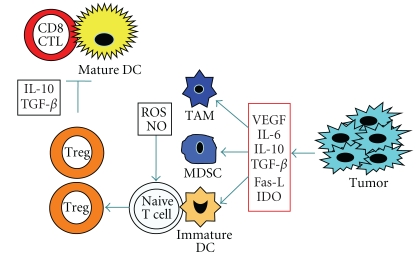
Immunosuppression in tumor microenvironment. Tumors secrete various factors such as VEGF, IL-6, IL-10, TGF- *β*, Fas-L, and IDO, all of which promote the accumulation of heterogeneous populations of tumor-associated macrophages (TAMs), myeloid-derived suppressor cells (MDSCs), or immature DCs. These immunosuppressive cells inhibit antitumor immunity by various mechanisms, including depletion of arginine and elaboration of reactive oxygen species (ROS) and nitrogen oxide (NO). The tumor microenvironment also promotes the accumulation of regulatory T cells (Tregs) that suppress CD8+ CTL function through secretion of IL-10 or TGF-*β* from Tregs and tumors.

Tumor/DC fusions aimed for inducing efficient antitumor immunity have provided important proofs of principle in both murine models and preclinical human models. However, immunological responses by DC/tumor fusions vaccine have not been associated with significant clinical responses. A major reason of the diversity is immunosuppressive microenvironment within the tumor. The microenvironment in solid tumors is consisted of tumor cells and stroma cells such as cancer-associated fibroblasts (CAFs), tolerogenic DCs, myeloid-derived suppressor cells (MDSCs), immunosuppressive tumor-associated macrophages (TAMs), and Tregs [66, 146–149] (Figure 3). Tumor cells and CAFs produce immunosuppressive substances such as vascular endothelial growth factor (VEGF) [150], IL-6 [151], IL-10 [151], transforming growth factor-*β* (TGF-*β*) [152], soluble Fas ligand (Fas-L) [153], and indolamine-2,3-dioxygenase (IDO) [154]. Tolerogenic DCs express low levels of MHC class I, II, and costimulatory molecules and produce increased levels of TGF-*β*, all of which are associated with generation of Tregs [155–157]. MDSCs suppress the activation of CD4+ and CD8+ T cells [158, 159] and also facilitate the generation of tumor-specific Tregs [160, 161]. TAMs promote tumor progression by generation of Tregs [162] and abolish tumor-specific CTLs [163]. As the results, generation of Tregs evades the antitumor immunity [164]. Indeed, an increase of Tregs population has been observed in the peripheral blood from patients with advanced cancer [165, 166] and is inversely related to the outcome of several human cancer treatments [167, 168]. Therefore, tumor/DC vaccines that struggle against the tumors with CTLs as well as depletion of Tregs may tip the balance in favor of immunostimulation.

## 8. Activation or Inactivation of Antitumor Immunity by Tumor/DC Fusions

**Figure 4:**
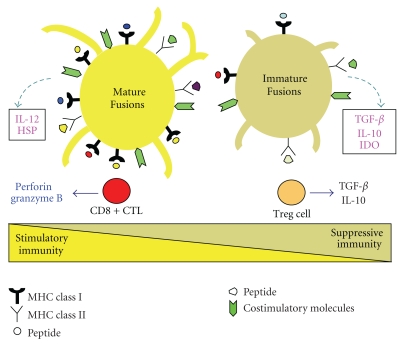
Activation or inactivation of T cells by tumor/DC fusions. After acquired antigens in the periphery, tumor/DC fusions migrate to the draining lymph nodes, where they encounter a cognate CD4+ or CD8+ T cells. The mature tumor/DC fusions produce stimulatory factors, such as IL-12 and heat-shock proteins (HSPs), while the immature fusions produce suppressive factors (TGF- *β*, IL-10, or IDO, etc.). High expression of costimulatory and MHC class I and II molecules by mature fusions is essential to promote survival and proliferative capacity of the activated CD8+ CTLs. Mature fusions induce efficient CD8+ T-cell activation with high production of perforin and granzyme B. On the other hand, immature fusions may induce, at least in part, Tregs. In tumor microenvironment, the consequence of products from tumor cells enhances local suppressive immunity.

Progress in antitumor immunotherapy has been aided by advances in the understanding of antigen presentation by DCs and the rules for governing polarization of subsequent immune responses toward CD4+ (Th1/Th2 phenotypes) or CD8+ T cells [2]. Importantly, the immunosuppressive microenvironment in tumors evades CTL responses during their induction and effector phase [165, 166]. Indeed, in cancer patients vaccinated with tumor/DC fusions, soluble factors derived from tumor cells inhibited the induction of CTL responses and promoted the generation of Tregs with immunosuppressive capacities [118]. One way to improve the CTL induction phase may be blockade of the negative soluble factors from tumor/DC fusions. In murine model, tumor-derived TGF-*β* reduced the efficacy of tumor/DC fusions vaccine via an in vivo mechanism [55]. However, the reduction of TGF-*β* derived from fusions inhibited Tregs generation and enhanced antitumor immunity [66]. Therefore, attention to these immunological bottlenecks may prove critical to fully harness the therapeutic potential of the fusions vaccine. Another approach for blocking the suppressive soluble factors from fusions is the use of adjuvants. The recognition of microbes by innate immune cells initiates activation of the whole immune system [169]. Toll-like receptors (TLRs) recognize various components of invading pathogens. It has been reported that DCs maturation by microbial products through TLRs is essential for abrogating the activity of Tregs in induction phase of T cells [170]. Moreover, crosspriming by DCs is based on the transfer of proteasome substrates that are transcriptionally upregulated by heat treatment in human tumor cells [171]. Therefore, we have generated mature fusions by fusing DCs stimulated with the TLR agonists and heat-treated tumor cells [100, 101]. The mature fusions had potent APC functions in induction phase of T cells, as demonstrated by (1) upregulation of multiple heat-shock proteins (HSPs), MHC class I and II, TAAs, CD80, CD86, CD83, and IL-12; (2) activation of CD4+ and CD8+ T cells able to produce IFN-*γ* at higher levels; (3) potent induction of cytotoxic activity specific for TAAs (CEA and MUC1) against tumors. Incorporating heat-treated tumor cells and TLR stimulated-DCs may increase the immunogenicity of tumor/DC fusions in induction of CTL responses. Similar results were also obtained from fusions generated with gastric cancer patients [172]. Immature fusions may stimulate a mixed T cell response characterized by the expansion of both CTL and Treg populations [109]. In addition, tumor/DC fusions activated by TLR agonists, IL-12, and anti-CD3/CD28 preferentially limited the generation of Tregs and promoted expansion of activated CTLs [109, 110]. Therefore, mature fusions have more active to stimulate CTL responses in the immunosuppressive environment in the growing tumor burden (Figure 4). Indeed, in murine models, tumor/DC fusions coadministrated with TLR9 (synthetic oligodeoxynucleotides (ODNs) containing specific bacterial unmethylated CpG motifs (CpG ODNs)) and TLR3 agonists (Poly(I:C)) significantly reduced melanoma metastasis through IL-12 production, compared with fusions alone [59, 82]. Moreover, tumor/DC fusions transduced with IL-12 [30, 87, 91, 96], IL-18 [90, 96], GM-CSF [47], IL-4 [88], CD40L [89] genes induced potentially increased therapeutic efficacy. 

Another approach designed to improve the efficacy of cancer vaccine is HSP70-based vaccine using tumor/DC fusion technology. The HSP70/peptide complexes (HSP70.PC) derived from tumor/DC fusions were especially different from those derived from tumor cells in enhanced association with immunologic peptides in animal models [173] and human models [172, 174]. HSP70.PC from human fusions induced T cells that expressed higher levels of IFN-*γ* and exhibited increased levels of killing of tumor cells, compared with those induced by HSP70.PC derived from tumor cells [172, 174]. Moreover, enhanced immunogenicity of HSP70.PC from fusions was associated with improved composition of the vaccine.

## 9. Combination of Treg Blockade and Tumor/DC Fusions Vaccine

Cancer vaccines must include some strategies to regulate the immunosuppressive cell types and tumor byproducts. Even if tumor/DC fusions were activated by TLRs, Tregs were not a little induced [105, 109, 118]. As Tregs is one of major obstacles for therapeutic cancer vaccines, depletion or blockade of Tregs might enhance rejection of endogenous immune-escaped tumor and improve tumor immunity. In most patients with melanoma (90%), recombinant IL-2-diphteria toxin fusion protein (ONTAK) treatment resulted in depletion of Tregs and sufficient induction of melanoma-specific CTL responses [175, 176]. Moreover, CTL-associated antigen-4 (CTLA-4) antagonistic antibodies also release a key negative regulatory pathway on T cells and enhance antitumor immunity [177–179]. Other antibodies, such as CD137 (4-1BB) [180], CD40 [181], and programmed death-1 (PD-1) [182] antagonists are currently investigated in various stages of preclinical and clinical development. In tumor/DC fusions approach, it has been reported that the fusions coadministrated with Treg depletion by anti-CD25 antibody enhanced the efficacy of immunotherapy in murine pancreatic models [68]. Therefore, a combination of control of Tregs and concomitant vaccination of mature tumor/DCs fusions may be a more promising approach for the induction of therapeutic antitumor immunity in patients with advanced cancer. 

Recently, to overcome negatively regulated pathway by Tregs, a combination therapy of vaccine and chemotherapy has been designed to counteract this immune suppression. For example, when adoptive immunotherapy was combined with nonmyeloablative lymphodepleting chemotherapy, 18 (51%) of 35 treated patients with refractory metastatic melanoma experienced objective clinical responses including three ongoing complete responses and 15 partial responses [183]. This improvement of clinical responses is most likely owing to the elimination of MDSCs and Tregs. Indeed, cytotoxic chemotherapy not only affects the tumor but also depletes MDSCs and Tregs [184]. Postchemotherapy immune system reconstitution may provide a unique opportunity for therapeutic intervention by shaping the repertoire towards responses to tumor antigens [147, 185, 186].

## 10. Summary

Although immunological responses have been observed in patients with advanced stage of cancer after being vaccinated with DC-based vaccines including tumor/DC fusions, the clinical responses are not as vigorous as in the animal models. Several aspects of cancer vaccines require the reduction of Tregs networks or suppressive tumor-microenvironments that inhibit the function of antitumor immune responses. To date, most of clinical trials have been enrolled patients who are in the advanced stages of cancer, which may have limited the clinical effectiveness because such individuals may not be able to mount an effective immune response. As tumor/DC fusions vaccine has been established as safe in phase I/II trials, the fusions vaccine should be tested in patients with early stage of cancer. Importantly, a combination therapy of cancer vaccines and other therapies such as conventional chemotherapy should be a more promising approach.

##  Conflict of Interests

The authors have no relevant affiliations or financial involvement with any organization or entity with a financial interest in or financial conflict with the subject matter or materials discussed in the paper.

## Figures and Tables

**Table 1:** Asessment of clinical trials by tumor/DC fusions-based vaccine.

	Tumor/DC Fusions			Patient	Clinical	
Tumor	Tumor Cells	Dendritic Cells	Coadministration	Number	Responses	Ref.
Melanoma	Autologous	Allogeneic		16	1 (CR)	[135]
					1 (PR)	[136]
					5 (SD)	
					9 (PD)	
	Autologous	Autologous		17	1 (PR)	[137]
					1 (SD)	
					15 (PD)	
	Autologous	Allogeneic		13	8 (SD)	[138]
					3 (SD)	
					2 (N)	
	Autologous	Autologous	rh IL-12	4	4 (PD)	[23]
	Autologous	Allogeneic	rh IL-2	11	1 (SD)	[139]
					10 (PD)	

Glioma	Autologous	Autologous		8	2 (PR)	[23]
					1 (SD)	
					5 (PD)	
	Autologous	Autologous	rh IL-12	12	3 (PR)	[24]
					2 (MR)	
					4 (SD)	
					3 (PD)	

Renal cell carcinoma	Autologous	Allogeneic		22	14 (SD)	[138]
					2 (PD)	
					3 (OR)	
					3 (N)	
	Autologous	Autologous		13	5 (SD)	[142]
					8 (PD)	
	Autologous	Allogeneic		20	2 (PR)	[143]
					8 (SD)	
					10 (PD)	
	Allogeneic	Allogeneic		8	3 (SD)	[144]
					5 (PD)	
	Autologous	Allogeneic		4	1 (SD)	[144]
					3 (PD)	
	Autologous	Allogeneic		10	1 (PR)	[145]
					6 (SD)	
					3 (PD)	

Breast cancer	Autologous	Autologous		10	2 (PR)	[142]
					1 (SD)	
					7 (PD)	
	Autologous	Autologous	rh IL-12	2	1 (SD)	[24]
					1 (PD)	

Gastric/Colorectal cancer	Autologous	Autologous	rh IL-12	3	1 (SD)	[24]
					2 (PD)	

Hepatocellular carcinoma	Autologous	Autologous		1	1 (PD)	[118]

Ovarian cancer	Autologous	Autologous	rh IL-12	3	2 (SD)	[24]
					1 (PD)	

CR: complete response; PR: partial response; MR: mixed response; SD: stable disease; PD: progressive disease.

OR: objective response; N: not evaluated.

## References

[1] Banchereau J, Steinman RM (1998). Dendritic cells and the control of immunity. *Nature*.

[2] Steinman RM (1991). The dendritic cell system and its role in immunogenicity. *Annual Review of Immunology*.

[3] Toes REM, Ossendorp F, Offringa R, Melief CJM (1999). CD4 T cells and their role in antitumor immune responses. *Journal of Experimental Medicine*.

[4] Shevach EM (2009). Mechanisms of foxp3+ T regulatory cell-mediated suppression. *Immunity*.

[5] Sakaguchi S, Yamaguchi T, Nomura T, Ono M (2008). Regulatory T cells and immune tolerance. *Cell*.

[6] Ziegler SF (2006). FOXP3: of mice and men. *Annual Review of Immunology*.

[7] Mougiakakos D, Choudhury A, Lladser A, Kiessling R, Johansson CC (2010). Regulatory T cells in cancer. *Advances in Cancer Research*.

[8] Banchereau J, Palucka AK (2005). Dendritic cells as therapeutic vaccines against cancer. *Nature Reviews Immunology*.

[9] Inaba K, Witmer-Pack M, Inaba M (1994). The tissue distribution of the B7-2 costimulator in mice: abundant expression on dendritic cells in situ and during maturation in vitro. *Journal of Experimental Medicine*.

[10] Young JW, Inaba K (1996). Dendritic cells as adjuvants for class I major histocompatibility complex-restricted antitumor immunity. *Journal of Experimental Medicine*.

[11] Inaba K, Pack M, Inaba M, Sakuta H, Isdell F, Steinman RM (1997). High levels of a major histocompatibility complex II-self peptide complex on dendritic cells from the T cell areas of lymph nodes. *Journal of Experimental Medicine*.

[12] Théry C, Amigorena S (2001). The cell biology of antigen presentation in dendritic cells. *Current Opinion in Immunology*.

[13] Celluzzi CM, Mayordomo JI, Storkus WJ, Lotze MT, Falo LD (1996). Peptide-pulsed dendritic cells induce antigen-specific, CTL-mediated protective tumor immunity. *Journal of Experimental Medicine*.

[14] Nestle FO, Alijagic S, Gilliet M (1998). Vaccination of melanoma patients with peptide- or tumor lysate-pulsed dendritic cells. *Nature Medicine*.

[15] Mayordomo JI, Zorina T, Storkus WJ (1995). Bone marrow-derived dendritic cells pulsed with synthetic tumour peptides elicit protective and therapeutic antitumour immunity. *Nature Medicine*.

[16] Gong J, Chen L, Chen D (1997). Induction of antigen-specific antitumor immunity with adenovirus-transduced dendritic cells. *Gene Therapy*.

[17] Koido S, Kashiwaba M, Chen D, Gendler S, Kufe D, Gong J (2000). Induction of antitumor immunity by vaccination of dendritic cells transfected with MUC1 RNA. *Journal of Immunology*.

[18] Gong J, Chen D, Kashiwaba M, Kufe D (1997). Induction of antitumor activity by immunization with fusions of dendritic and carcinoma cells. *Nature Medicine*.

[19] Gong J, Koido S, Calderwood SK (2008). Cell fusion: from hybridoma to dendritic cell-based vaccine. *Expert Review of Vaccines*.

[20] Koido S, Hara E, Homma S, Fujise K, Gong J, Tajiri H (2007). Dendritic/tumor fusion cell-based vaccination against cancer. *Archivum Immunologiae et Therapiae Experimentalis*.

[21] Koido S, Hara E, Homma S (2009). Cancer vaccine by fusions of dendritic and cancer cells. *Clinical and Developmental Immunology*.

[22] Koido S, Hara E, Homma S, Ohkusa T, Gong J, Tajiri H (2009). Cancer immunotherapy by fusions of dendritic cells and tumor cells. *Immunotherapy*.

[23] Homma S, Kikuchi T, Ishiji N (2005). Cancer immunotherapy by fusions of dendritic and tumour cells and rh-IL-12. *European Journal of Clinical Investigation*.

[24] Kikuchi T, Akasaki Y, Abe T (2004). Vaccination of glioma patients with fusions of dendritic and glioma cells and recombinant human interleukin 12. *Journal of Immunotherapy*.

[25] Koido S, Hara E, Torii A (2005). Induction of antigen-specific CD4- and CD8-mediated T-cell responses by fusions of autologous dendritic cells and metastatic colorectal cancer cells. *International Journal of Cancer*.

[26] Wang J, Saffold S, Cao X, Krauss J, Chen W (1998). Eliciting T cell immunity against poorly immunogenic tumors by immunization with dendritic cell-tumor fusion vaccines. *Journal of Immunology*.

[27] Hayashi T, Tanaka H, Tanaka J (2002). Immunogenicity and therapeutic efficacy of dendritic-tumor hybrid cells generated by electrofusion. *Clinical Immunology*.

[28] Tanaka H, Shimizu K, Hayashi T, Shu S (2002). Therapeutic immune response induced by electrofusion of dendritic and tumor cells. *Cellular Immunology*.

[29] Jantscheff P, Spagnoli G, Zajac P, Rochlitz C (2002). Cell fusion: an approach to generating constitutively proliferating human tumor antigen-presenting cells. *Cancer Immunology, Immunotherapy*.

[30] Suzuki T, Fukuhara T, Tanaka M (2005). Vaccination of dendritic cells loaded with interleukin-12-secreting cancer cells augments in vivo antitumor immunity: characteristics of syngeneic and allogeneic antigen-presenting cell cancer hybrid cells. *Clinical Cancer Research*.

[31] Shimizu K, Kuriyama H, Kjaergaard J, Lee W, Tanaka H, Shu S (2004). Comparative analysis of antigen loading strategies of dendritic cells for tumor immunotherapy. *Journal of Immunotherapy*.

[32] Phan V, Errington F, Cheong SC (2003). A new genetic method to generate and isolate small, short-lived but highly potent dendritic cell-tumor cell hybrid vaccines. *Nature Medicine*.

[33] Hiraoka K, Yamamoto S, Otsuru S (2004). Enhanced tumor-specific long-term immunity of hemaggluttinating virus of Japan-mediated dendritic cell-tumor fused cell vaccination by coadministration with CpG oligodeoxynucleotides. *Journal of Immunology*.

[34] Koido S, Ohana M, Liu C (2004). Dendritic cells fused with human cancer cells: morphology, antigen expression, and T cell stimulation. *Clinical Immunology*.

[35] Steinman RM, Swanson J (1995). The endocytic activity of dendritic cells. *Journal of Experimental Medicine*.

[36] Watts C (2004). The exogenous pathway for antigen presentation on major histocompatibility complex class II and CD1 molecules. *Nature Immunology*.

[37] Berard F, Blanco P, Davoust J (2000). Cross-priming of naive CD8 T cells against melanoma antigens using dendritic cells loaded with killed allogeneic melanoma cells. *Journal of Experimental Medicine*.

[38] Heath WR, Carbone FR (2001). Cross-presentation, dendritic cells, tolerance and immunity. *Annual Review of Immunology*.

[39] Wolkers MC, Brouwenstijn N, Bakker AH, Toebes M, Schumacher TNM (2004). Antigen bias in T cell cross-priming. *Science*.

[40] Benencia F, Courrèges MC, Coukos G (2008). Whole tumor antigen vaccination using dendritic cells: comparison of RNA electroporation and pulsing with UV-irradiated tumor cells. *Journal of Translational Medicine*.

[41] Shu S, Zheng R, Lee WT, Cohen PA (2007). Immunogenicity of dendritic-tumor fusion hybrids and their utility in cancer immunotherapy. *Critical Reviews in Immunology*.

[42] Finn OJ (2008). Cancer immunology. *The New England Journal of Medicine*.

[43] Gong J, Avigan D, Chen D (2000). Activation of antitumor cytotoxic T lymphocytes by fusions of human dendritic cells and breast carcinoma cells. *Proceedings of the National Academy of Sciences of the United States of America*.

[44] Gong J, Nikrui N, Chen D (2000). Fusions of human ovarian carcinoma cells with autologous or allogeneic dendritic cells induce antitumor immunity. *Journal of Immunology*.

[45] Koido S, Tanaka Y, Chen D, Kufe D, Gong J (2002). The kinetics of in vivo priming of CD4 and CD8 T cells by dendritic/tumor fusion cells in MUC1-transgenic mice. *Journal of Immunology*.

[46] Tanaka Y, Koido S, Ohana M, Liu C, Gong J (2005). Induction of impaired antitumor immunity by fusion of MHC class II-deficient dendritic cells with tumor cells. *Journal of Immunology*.

[47] Cao X, Zhang W, Wang J (1999). Therapy of established tumour with a hybrid cellular vaccine generated by using granulocyte-macrophage colony-stimulating factor genetically modified dendritic cells. *Immunology*.

[48] Li J, Holmes LM, Franek KJ, Burgin KE, Wagner TE, Wei Y (2001). Purified hybrid cells from dendritic cell and tumor cell fusions are superior activators of antitumor immunity. *Cancer Immunology, Immunotherapy*.

[49] Kuriyama H, Watanabe S, Kjaergaard J (2006). Mechanism of third signals provided by IL-12 and OX-40R ligation in eliciting therapeutic immunity following dendritic-tumor fusion vaccination. *Cellular Immunology*.

[50] Ishida A, Tanaka H, Hiura T (2007). Generation of anti-tumour effector T cells from naive T cells by stimulation with dendritic/tumour fusion cells. *Scandinavian Journal of Immunology*.

[51] Ko E, Luo W, Peng L, Wang X, Ferrone S (2007). Mouse dendritic-endothelial cell hybrids and 4-1BB costimulation elicit antitumor effects mediated by broad antiangiogenic immunity. *Cancer Research*.

[52] Šalomskaite-Davalgiene S, Čepurniene K, Šatkauskas S, Venslauskas MS, Mir LM (2009). Extent of cell electrofusion in vitro and in vivo is cell line dependent. *Anticancer Research*.

[53] Gong J, Apostolopoulos V, Chen D (2000). Selection and characterization of MUC1-specific CD8+ T cells from MUC1 transgenic mice immunized with dendritic-carcinoma fusion cells. *Immunology*.

[54] Kao JY, Gong Y, Chen C-M, Zheng Q-D, Chen J-J (2003). Tumor-derived TGF-*β* reduces the efficacy of dendritic cell/tumor fusion vaccine. *Journal of Immunology*.

[55] Iinuma T, Homma S, Noda T, Kufe D, Ohno T, Toda G (2004). Prevention of gastrointestinal tumors based on adenomatous polyposis coli gene mutation by dendritic cell vaccine. *Journal of Clinical Investigation*.

[56] Kao JY, Zhang M, Chen C-M, Chen J-J (2005). Superior efficacy of dendritic cell-tumor fusion vaccine compared with tumor lysate-pulsed dendritic cell vaccine in colon cancer. *Immunology Letters*.

[57] Xu F, Ye Y-J, Cui Z-R, Wang S (2005). Allogeneic dendritomas induce anti-tumour immunity against metastatic colon cancer. *Scandinavian Journal of Immunology*.

[58] Yasuda T, Kamigaki T, Kawasaki K (2007). Superior anti-tumor protection and therapeutic efficacy of vaccination with allogeneic and semiallogeneic dendritic cell/tumor cell fusion hybrids for murine colon adenocarcinoma. *Cancer Immunology, Immunotherapy*.

[59] Cho EI, Tan C, Koski GK, Cohen PA, Shu S, Lee WT (2010). Toll-like receptor agonists as third signals for dendritic cell-tumor fusion vaccines. *Head and Neck*.

[60] Gong J, Chen D, Kashiwaba M (1998). Reversal of tolerance to human MUC1 antigen in MUC1 transgenic mice immunized with fusions of dendritic and carcinoma cells. *Proceedings of the National Academy of Sciences of the United States of America*.

[61] Lindner M, Schirrmacher V (2002). Tumour cell-dendritic cell fusion for cancer immunotherapy: comparison of therapeutic efficiency of polyethylen-glycol versus electro-fusion protocols. *European Journal of Clinical Investigation*.

[62] Xia J, Tanaka Y, Koido S (2003). Prevention of spontaneous breast carcinoma by prophylactic vaccination with dendritic/tumor fusion cells. *Journal of Immunology*.

[63] Chen D, Xia J, Tanaka Y (2003). Immunotherapy of spontaneous mammary carcinoma with fusions of dendritic cells and mucin 1-positive carcinoma cells. *Immunology*.

[64] Tamai H, Watanabe S, Zheng R (2008). Effective treatment of spontaneous metastases derived from a poorly immunogenic murine mammary carcinoma by combined dendritic-tumor hybrid vaccination and adoptive transfer of sensitized T cells. *Clinical Immunology*.

[65] Zhang M, Berndt BE, Chen J-J, Kao JY (2008). Expression of a soluble TGF-*β* receptor by tumor cells enhances dendritic cell/tumor fusion vaccine efficacy. *Journal of Immunology*.

[66] Guo G-H, Chen S-Z, Yu J (2008). In vivo anti-tumor effect of hybrid vaccine of dendritic cells and esophageal carcinoma cells on esophageal carcinoma cell line 109 in mice with severe combined immune deficiency. *World Journal of Gastroenterology*.

[67] Ziske C, Etzrodt PE, Eliu A-S (2009). Increase of in vivo antitumoral activity by CD40L (CD154) gene transfer into pancreatic tumor cell-dendritic cell hybrids. *Pancreas*.

[68] Yamamoto M, Kamigaki T, Yamashita K (2009). Enhancement of anti-tumor immunity by high levels of Th1 and Th17 with a combination of dendritic cell fusion hybrids and regulatory T cell depletion in pancreatic cancer. *Oncology Reports*.

[69] Homma S, Toda G, Gong J, Kufe D, Ohno T (2001). Preventive antitumor activity against hepatocellular carcinoma (HCC) induced by immunization with fusions of dendritic cells and HCC cells in mice. *Journal of Gastroenterology*.

[70] Zhang J-K, Li J, Zhang J, Chen H-B, Chen S-B (2003). Antitumor immunopreventive and immunotherapeutic effect in mice induced by hybrid vaccine of dendritic cells and hepatocarcinoma in vivo. *World Journal of Gastroenterology*.

[71] Iriei M, Homma S, Komita H (2004). Inhibition of spontaneous development of liver tumors by inoculation with dendritic cells loaded with hepatocellular carcinoma cells in C3H/HeNCRJ mice. *International Journal of Cancer*.

[72] Zhang H-M, Zhang L-W, Liu W-C, Cheng J, Si X-M, Ren J (2006). Comparative analysis of DC fused with tumor cells or transfected with tumor total RNA as potential cancer vaccines against hepatocellular carcinoma. *Cytotherapy*.

[73] Sheng X-L, Zhang H (2007). In-vitro activation of cytotoxic T lymphocytes by fusion of mouse hepatocellular carcinoma cells and lymphotactin gene-modified dendritic cells. *World Journal of Gastroenterology*.

[74] Celluzzi CM, Falo LD (1998). Gutting edge: physical interaction between dendritic cells and tumor cells results in an immunogen that induces protective and therapeutic tumor rejection. *Journal of Immunology*.

[75] Šímová J, Bubeník J, Bieblová J, Indrová M, Jandlová T (2005). Immunotherapeutic efficacy of vaccines generated by fusion of dendritic cells and HPV16-associated tumour cells. *Folia Biologica*.

[76] Savai R, Schermuly RT, Schneider M (2006). Hybrid-primed lymphocytes and hybrid vaccination prevent tumor growth of Lewis lung carcinoma in mice. *Journal of Immunotherapy*.

[77] Savai R, Schermuly RT, Pullamsetti SS (2007). A combination hybrid-based vaccination/adoptive cellular therapy to prevent tumor growth by involvement of T cells. *Cancer Research*.

[78] Ou X, Cai S, Liu P (2008). Enhancement of dendritic cell-tumor fusion vaccine potency by indoleamine-pyrrole 2,3-dioxygenase inhibitor, 1-MT. *Journal of Cancer Research and Clinical Oncology*.

[79] Siders WM, Vergilis KL, Johnson C, Shields J, Kaplan JM (2003). Induction of specific antitumor immunity in the mouse with the electrofusion product of tumor cells and dendritic cells. *Molecular Therapy*.

[80] Zheng R, Cohen PA, Paustian CA (2008). Paired toll-like receptor agonists enhance vaccine therapy through induction of interleukin-12. *Cancer Research*.

[81] Kjaergaard J, Shimizu K, Shu S (2003). Electrofusion of syngeneic dendritic cells and tumor generates potent therapeutic vaccine. *Cellular Immunology*.

[82] Matsue H, Matsue K, Edelbaum D, Walters M, Morita A, Takashima A (2004). New strategy for efficient selection of dendritic cell-tumor hybrids and clonal heterogeneity of resulting hybrids. *Cancer Biology and Therapy*.

[83] Kim G-Y, Chae H-J, Kim K-H (2007). Dendritic cell-tumor fusion vaccine prevents tumor growth in vivo. *Bioscience, Biotechnology and Biochemistry*.

[84] Yu Z, Ma B, Zhou Y (2007). Allogeneic tumor vaccine produced by electrofusion between osteosarcoma cell line and dendritic cells in the induction of antitumor immunity. *Cancer Investigation*.

[85] Yanai S, Adachi Y, Fuijisawa J-I (2009). Anti-tumor effects of fusion cells of type 1 dendritic cells and Meth A tumor cells using hemagglutinating virus of Japan-envelope. *International Journal of Oncology*.

[86] Gong J, Koido S, Chen D (2002). Immunization against murine multiple myeloma with fusions of dendritic and plasmacytoma cells is potentiated by interleukin 12. *Blood*.

[87] Zhang W, Yang H, Zeng H (2002). Enhancing antitumor by immunization with fusion of dendritic cells and engineered tumor cells. *Journal of Huazhong University of Science and Technology—Medical Science*.

[88] Liu Y, Zhang W, Chan T, Saxena A, Xiang J (2002). Engineered fusion hybrid vaccine of IL-4 gene-modified myeloma and relative mature dendritic cells enhances antitumor immunity. *Leukemia Research*.

[89] Hao S, Bi X, Xu S (2004). Enhanced antitumor immunity derived from a novel vaccine of fusion hybrid between dendritic and engineered myeloma cells. *Experimental Oncology*.

[90] Xia D, Li F, Xiang J (2004). Engineered fusion hybrid vaccine of IL-18 gene-modified tumor cells and dendritic cells induces enhanced antitumor immunity. *Cancer Biotherapy and Radiopharmaceuticals*.

[91] Shi M, Su L, Hao S, Guo X, Xiang J (2005). Fusion hybrid of dendritic cells and engineered tumor cells expressing interleukin-12 induces type 1 immune responses against tumor. *Tumori*.

[92] Quéant S, Sarde C-O, Gobert M-G, Kadouche J, Roseto A (2005). Antitumor response against myeloma cells by immunization with mouse syngenic dendritoma. *Hybridoma*.

[93] Alvarez E, Moga E, Barquinero J, Sierra J, Briones J (2010). Dendritic and tumor cell fusions transduced with adenovirus encoding CD40L eradicate B-cell lymphoma and induce a Th17-type response. *Gene Therapy*.

[94] Lespagnard L, Mettens’ P, Verheyden A-M (1998). Dendritic cells fused with mastocytoma cells elicit therapeutic antitumor immunity. *International Journal of Cancer*.

[95] Wells JW, Cowled CJ, Darling D (2007). Semi-allogeneic dendritic cells can induce antigen-specific T-cell activation, which is not enhanced by concurrent alloreactivity. *Cancer Immunology, Immunotherapy*.

[96] Iinuma H, Okinaga K, Fukushima R (2006). Superior protective and therapeutic effects of IL-12 and IL-18 gene-transduced dendritic neuroblastoma fusion cells on liver metastasis of murine neuroblastoma. *Journal of Immunology*.

[97] Draube A, Beyer M, Schumer S (2007). Efficient activation of autologous tumor-specific T cells: a simple coculture technique of autologous dendritic cells compared to established cell fusion strategies in primary human colorectal carcinoma. *Journal of Immunotherapy*.

[98] Koido S, Hara E, Homma S (2005). Dendritic cells fused with allogeneic colorectal cancer cell line present multiple colorectal cancer-specific antigens and induce antitumor immunity against autologous tumor cells. *Clinical Cancer Research*.

[99] Hock BD, Roberts G, McKenzie JL (2005). Exposure to the electrofusion process can increase the immunogenicity of human cells. *Cancer Immunology, Immunotherapy*.

[100] Koido S, Hara E, Homma S (2007). Streptococcal preparation OK-432 promotes fusion efficiency and enhances induction of antigen-specific CTL by fusions of dendritic cells and colorectal cancer cells. *Journal of Immunology*.

[101] Koido S, Hara E, Homma S (2007). Synergistic induction of antigen-specific CTL by fusions of TLR-stimulated dendritic cells and heat-stressed tumor cells. *Journal of Immunology*.

[102] Yang J-Y, Cao D-Y, Ma L-Y, Liu W-C (2010). Dendritic cells fused with allogeneic hepatocellular carcinoma cell line compared with fused autologous tumor cells as hepatocellular carcinoma vaccines. *Hepatology Research*.

[103] Imura K, Ueda Y, Hayashi T (2006). Induction of cytotoxic T lymphocytes against human cancer cell lines using dendritic cell-tumor cell hybrids generated by a newly developed electrofusion technique. *International Journal of Oncology*.

[104] Matsumoto S, Saito H, Tsujitani S, Ikeguchi M (2006). Allogeneic gastric cancer cell-dendritic cell hybrids induce tumor antigen (carcinoembryonic antigen) specific CD8+ T cells. *Cancer Immunology, Immunotherapy*.

[105] Koido S, Hara E, Homma S (2010). Dendritic/pancreatic carcinoma fusions for clinical use: comparative functional analysis of healthy- versus patient-derived fusions. *Clinical Immunology*.

[106] Zhang Y, Ma B, Zhou Y (2007). Dendritic cells fused with allogeneic breast cancer cell line induce tumor antigen-specific CTL responses against autologous breast cancer cells. *Breast Cancer Research and Treatment*.

[107] Serhal K, Baillou C, Ghinea N (2007). Characteristics of hybrid cells obtained by dendritic cell/tumour cell fusion in a T-47D breast cancer cell line model indicate their potential as anti-tumour vaccines. *International Journal of Oncology*.

[108] Koido S, Tanaka Y, Tajiri H, Gong J (2007). Generation and functional assessment of antigen-specific T cells stimulated by fusions of dendritic cells and allogeneic breast cancer cells. *Vaccine*.

[109] Vasir B, Wu Z, Crawford K (2008). Fusions of dendritic cells with breast carcinoma stimulate the expansion of regulatory T cells while concomitant exposure to IL-12, CpG oligodeoxynucleotides, and Anti-CD3/CD28 promotes the expansion of activated tumor reactive cells. *Journal of Immunology*.

[110] Rosenblatt J, Wu Z, Vasir B (2010). Generation of tumor-specific t lymphocytes using dendritic cell/tumor fusions and anti-CD3/CD28. *Journal of Immunotherapy*.

[111] Weise JB, Maune S, Görögh T (2004). A dendritic cell based hybrid cell vaccine generated by electrofusion for immunotherapy strategies in HNSCC. *Auris Nasus Larynx*.

[112] Koido S, Nikrui N, Ohana M (2005). Assessment of fusion cells from patient-derived ovarian carcinoma cells and dendritic cells as a vaccine for clinical use. *Gynecologic Oncology*.

[113] Cheong SC, Blangenois I, Franssen J-D (2006). Generation of cell hybrids via a fusogenic cell line. *Journal of Gene Medicine*.

[114] Lundqvist A, Palmborg A, Bidla G, Whelan M, Pandha H, Pisa P (2004). Allogeneic tumor-dendritic cell fusion vaccines for generation of broad prostate cancer T-cell responses. *Medical Oncology*.

[115] Kim T-B, Park HK, Chang JH (2010). The establishment of dendritic cell-tumor fusion vaccines for hormone refractory prostate cancer cell. *Korean Journal of Urology*.

[116] Gottfried E, Krieg R, Eichelberg C, Andreesen R, Mackensen A, Krause SW (2002). Characterization of cells prepared by dendritic cell-tumor cell fusion. *Cancer Immunity*.

[117] Hu Z, Liu S, Mai X, Hu Z, Liu C (2010). Anti-tumor effects of fusion vaccine prepared by renal cell carcinoma 786-O cell line and peripheral blood dendritic cells of healthy volunteers in vitro and in human immune reconstituted SCID mice. *Cellular Immunology*.

[118] Koido S, Homma S, Hara E (2008). In vitro generation of cytotoxic and regulatory T cells by fusions of human dendritic cells and hepatocellular carcinoma cells. *Journal of Translational Medicine*.

[119] Cao D-Y, Yang J-Y, Yue S-Q (2009). Comparative analysis of DC fused with allogeneic hepatocellular carcinoma cell line HepG2 and autologous tumor cells as potential cancer vaccines against hepatocellular carcinoma. *Cellular Immunology*.

[120] Xu F, Ye Y-J, Liu W, Kong M, He Y, Wang S (2010). Dendritic cell/tumor hybrids enhances therapeutic efficacy against colorectal cancer liver metastasis in SCID mice. *Scandinavian Journal of Gastroenterology*.

[121] Galea-Lauri J, Darling D, Mufti G, Harrison P, Farzaneh F (2002). Eliciting cytotoxic T lymphocytes against acute myeloid leukemia-derived antigens: evaluation of dendritic cell-leukemia cell hybrids and other antigen-loading strategies for dendritic cell-based vaccination. *Cancer Immunology, Immunotherapy*.

[122] Kokhaei P, Rezvany MR, Virving L (2003). Dendritic cells loaded with apoptotic tumour cells induce a stronger T-cell response than dendritic cell-tumour hybrids in B-CLL. *Leukemia*.

[123] Gong J, Koido S, Kato Y (2004). Induction of anti-leukemic cytotoxic T lymphocytes by fusion of patient-derived dendritic cells with autologous myeloblasts. *Leukemia Research*.

[124] Banat G-A, Usluoglu N, Hoeck M, Ihlow K, Hoppmann S, Pralle H (2004). Dendritic cells fused with core binding factor-beta positive acute myeloid leukaemia blast cells induce activation of cytotoxic lymphocytes. *British Journal of Haematology*.

[125] Allgeier T, Garhammer S, Nößner E (2007). Dendritic cell-based immunogens for B-cell chronic lymphocytic leukemia. *Cancer Letters*.

[126] Lei Z, Zhang G-M, Hong M, Feng Z-H, Huang B (2009). Fusion of dendritic cells and CD34+CD38-acute myeloid leukemia (AML) cells potentiates targeting AML-initiating cells by specific CTL induction. *Journal of Immunotherapy*.

[127] Raje N, Hideshima T, Davies FE (2004). Tumour cell/dendritic cell fusions as a vaccination strategy for multiple myeloma. *British Journal of Haematology*.

[128] Vasir B, Borges V, Wu Z (2005). Fusion of dendritic cells with multiple myeloma cells results in maturation and enhanced antigen presentation. *British Journal of Haematology*.

[129] Yu Z, Ma B, Zhou Y, Zhang M, Qiu X, Fan Q (2005). Activation of antitumor cytotoxic T lymphocytes by fusion of patient-derived dendritic cells with autologous osteosarcoma. *Experimental Oncology*.

[130] Guo W, Guo Y, Tang S, Qu H, Zhao H (2008). Dendritic cell-Ewing’s sarcoma cell hybrids enhance antitumor immunity. *Clinical Orthopaedics and Related Research*.

[131] Parkhurst MR, DePan C, Riley JP, Rosenberg SA, Shu S (2003). Hybrids of dendritic cells and tumor cells generated by electrofusion simultaneously present immunodominant epitopes from multiple human tumor-associated antigens in the context of MHC class I and class II molecules. *Journal of Immunology*.

[132] Trevor KT, Cover C, Ruiz YW (2004). Generation of dendritic cell-tumor cell hybrids by electrofusion for clinical vaccine application. *Cancer Immunology, Immunotherapy*.

[133] Neves AR, Ensina LFC, Anselmo LB (2005). Dendritic cells derived from metastatic cancer patients vaccinated with allogeneic dendritic cell-autologous tumor cell hybrids express more CD86 and induce higher levels of interferon-gamma in mixed lymphocyte reactions. *Cancer Immunology, Immunotherapy*.

[134] Sukhorukov VL, Reuss R, Endter JM (2006). A biophysical approach to the optimisation of dendritic-tumour cell electrofusion. *Biochemical and Biophysical Research Communications*.

[135] Trefzer U, Weingart G, Chen Y (2000). Hybrid cell vaccination for cancer immune therapy: first clinical trial with metastatic melanoma. *International Journal of Cancer*.

[136] Trefzer U, Herberth G, Wohlan K (2005). Tumour-dendritic hybrid cell vaccination for the treatment of patients with malignant melanoma: immunological effects and clinical results. *Vaccine*.

[137] Krause SW, Neumann C, Soruri A, Mayer S, Peters JH, Andreesen R (2002). The treatment of patients with disseminated malignant melanoma by vaccination with autologous cell hybrids of tumor cells and dendritic cells. *Journal of Immunotherapy*.

[138] Barbuto JAM, Ensina LFC, Neves AR (2004). Dendritic cell-tumor cell hybrid vaccination for metastatic cancer. *Cancer Immunology, Immunotherapy*.

[139] Haenssle HA, Krause SW, Emmert S (2004). Hybrid cell vaccination in metastatic melanoma: clinical and immunologic results of a phase I/II study. *Journal of Immunotherapy*.

[140] Kikuchi T, Akasaki Y, Irie M, Homma S, Abe T, Ohno T (2001). Results of a phase I clinical trial of vaccination of glioma patients with fusions of dendritic and glioma cells. *Cancer Immunology, Immunotherapy*.

[141] Homma S, Sagawa Y, Ito M, Ohno T, Toda G (2006). Cancer immunotherapy using dendritic/tumour-fusion vaccine induces elevation of serum anti-nuclear antibody with better clinical responses. *Clinical and Experimental Immunology*.

[142] Avigan D, Vasir B, Gong J (2004). Fusion cell vaccination of patients with metastatic breast and renal cancer induces immunological and clinical responses. *Clinical Cancer Research*.

[143] Avigan DE, Vasir B, George DJ (2007). Phase I/II study of vaccination with electrofused allogeneic dendritic cells/autologous tumor-derived cells in patients with stage IV renal cell carcinoma. *Journal of Immunotherapy*.

[144] Märten A, Renoth S, Heinicke T (2003). Allogeneic dendritic cells fused with tumor cells: preclinical results and outcome of a clinical phase I/II trial in patients with metastatic renal cell carcinoma. *Human Gene Therapy*.

[145] Zhou J, Weng D, Zhou F (2009). Patient-derived renal cell carcinoma cells fused with allogeneic dendritic cells elicit anti-tumor activity: in vitro results and clinical responses. *Cancer Immunology, Immunotherapy*.

[146] Jin P, Han TH, Ren J (2010). Molecular signatures of maturing dendritic cells: implications for testing the quality of dendritic cell therapies. *Journal of Translational Medicine*.

[147] Morse MA, Hall JR, Plate JMD (2009). Countering tumor-induced immunosuppression during immunotherapy for pancreatic cancer. *Expert Opinion on Biological Therapy*.

[148] Kormelink TG, Abudukelimu A, Redegeld FA (2009). Mast cells as target in cancer therapy. *Current Pharmaceutical Design*.

[149] Fassnacht M, Lee J, Milazzo C (2005). Induction of CD4+ and CD8+ T-cell responses to the human stromal antigen, fibroblast activation protein: implication for cancer immunotherapy. *Clinical Cancer Research*.

[150] Fricke I, Mirza N, Dupont J (2007). Vascular endothelial growth factor-trap overcomes defects in dendritic cell differentiation but does not improve antigen-specific immune responses. *Clinical Cancer Research*.

[151] Elgert KD, Alleva DG, Mullins DW (1998). Tumor-induced immune dysfunction: the macrophage connection. *Journal of Leukocyte Biology*.

[152] Teicher BA (2007). Transforming growth factor-*β* and the immune response to malignant disease. *Clinical Cancer Research*.

[153] Houston A, Bennett MW, O’Sullivan GC, Shanahan F, O’Connell J (2003). Fas ligand mediates immune privilege and not inflammation in human colon cancer, irrespective of TGF-*β* expression. *British Journal of Cancer*.

[154] Uyttenhove C, Pilotte L, Théate I (2003). Evidence for a tumoral immune resistance mechanism based on tryptophan degradation by indoleamine 2,3-dioxygenase. *Nature Medicine*.

[155] Veldhoen M, Moncrieffe H, Hocking RJ, Atkins CJ, Stockinger B (2006). Modulation of dendritic cell function by naive and regulatory CD4 + T cells. *Journal of Immunology*.

[156] Kushwah R, Wu J, Oliver JR (2010). Uptake of apoptotic DC converts immature DC into tolerogenic DC that induce differentiation of Foxp3+ Treg. *European Journal of Immunology*.

[157] Anderson MJ, Shafer-Weaver K, Greenberg NM, Hurwitz AA (2007). Tolerization of tumor-specific T cells despite efficient initial priming in a primary murine model of prostate cancer. *Journal of Immunology*.

[158] Ko HJ, Lee JM, Kim YJ, Kim YS, Lee KA, Kang CY (2009). Immunosuppressive myeloid-derived suppressor cells can be converted into immunogenic APCs with the help of activated NKT cells: an alternative cell-based antitumor vaccine. *Journal of Immunology*.

[159] Ochoa AC, Zea AH, Hernandez C, Rodriguez PC (2007). Arginase, prostaglandins, and myeloid-derived suppressor cells in renal cell carcinoma. *Clinical Cancer Research*.

[160] Pan P-Y, Ma G, Weber KJ (2010). Immune stimulatory receptor CD40 is required for T-cell suppression and T regulatory cell activation mediated by myeloid-derived suppressor cells in cancer. *Cancer Research*.

[161] Srivastava MK, Sinha P, Clements VK, Rodriguez P, Ostrand-Rosenberg S (2010). Myeloid-derived suppressor cells inhibit T-cell activation by depleting cystine and cysteine. *Cancer Research*.

[162] Zhou J, Ding T, Pan W, Zhu L-Y, Li A, Zheng L (2009). Increased intratumoral regulatory T cells are related to intratumoral macrophages and poor prognosis in hepatocellular carcinoma patients. *International Journal of Cancer*.

[163] Nagorsen D, Voigt S, Berg E, Stein H, Thiel E, Loddenkemper C (2007). Tumor-infiltrating macrophages and dendritic cells in human colorectal cancer: relation to local regulatory T cells, systemic T-cell response against tumor-associated antigens and survival. *Journal of Translational Medicine*.

[164] Liu VC, Wong LY, Jang T (2007). Tumor evasion of the immune system by converting CD4+CD25—T cells into CD4+CD25+ T regulatory cells: role of tumor-derived TGF-*β*. *Journal of Immunology*.

[165] Strauss L, Bergmann C, Gooding W, Johnson JT, Whiteside TL (2007). The frequency and suppressor function of CD4+CD25 highFoxp3+ T cells in the circulation of patients with squamous cell carcinoma of the head and neck. *Clinical Cancer Research*.

[166] Strauss L, Bergmann C, Whiteside TL (2007). Functional and phenotypic characteristics of CD4+CD25 highFoxp3+ Treg clones obtained from peripheral blood of patients with cancer. *International Journal of Cancer*.

[167] Liyanage UK, Moore TT, Joo H-G (2002). Prevalence of regulatory T cells is increased in peripheral blood and tumor microenvironment of patients with pancreas or breast adenocarcinoma. *Journal of Immunology*.

[168] Ichihara F, Kono K, Takahashi A, Kawaida H, Sugai H, Fujii H (2003). Increased populations of regulatory T cells in peripheral blood and tumor-infiltrating lymphocytes in patients with gastric and esophageal cancers. *Clinical Cancer Research*.

[169] Hemmi H, Akira S (2005). TLR signalling and the function of dendritic cells. *Chemical Immunology and Allergy*.

[170] Pasare C, Medzhitov R (2003). Toll pathway-dependent blockade of CD4+CD25+ T cell-mediated suppression by dendritic cells. *Science*.

[171] Callahan MK, Wohlfert EA, Ménoret A, Srivastava PK (2006). Heat shock up-regulates Imp2 and Imp7 and enhances presentation of immunoproteasome-dependent epitopes. *Journal of Immunology*.

[172] Koide T, Iinuma H, Fukushima R (2009). Efficient CTL productivity of modified fusion cells by increase of heat shock protein 70. *Oncology Reports*.

[173] Enomoto Y, Bharti A, Khaleque AA (2006). Enhanced immunogenicity of heat shock protein 70 peptide complexes from dendritic cell-tumor fusion cells. *Journal of Immunology*.

[174] Gong J, Zhang Y, Durfee J (2010). A heat shock protein 70-based vaccine with enhanced immunogenicity for clinical use. *Journal of Immunology*.

[175] Rech AJ, Vonderheide RH (2009). Clinical use of anti-CD25 antibody daclizumab to enhance immune responses to tumor antigen vaccination by targeting regulatory T cells. *Annals of the New York Academy of Sciences*.

[176] Mahnke K, Schönfeld K, Fondel S (2007). Depletion of CD4+CD25+ human regulatory T cells in vivo: kinetics of Treg depletion and alterations in immune functions in vivo and in vitro. *International Journal of Cancer*.

[177] Fong L, Small EJ (2008). Anti-cytotoxic T-lymphocyte antigen-4 antibody: the first in an emerging class of immunomodulatory antibodies for cancer treatment. *Journal of Clinical Oncology*.

[178] Ribas A, Comin-Anduix B, Chmielowski B (2009). Dendritic cell vaccination combined with CTLA4 blockade in patients with metastatic melanoma. *Clinical Cancer Research*.

[179] Saha A, Chatterjee SK (2010). Combination of CTL-associated antigen-4 blockade and depletion of CD25 + regulatory T cells enhance tumour immunity of dendritic cell-based vaccine in a mouse model of colon cancer. *Scandinavian Journal of Immunology*.

[180] Sharma RK, Elpek KG, Yolcu ES (2009). Costimulation as a platform for the development of vaccines: a peptide-based vaccine containing a novel form of 4-1BB ligand eradicates established tumors. *Cancer Research*.

[181] Ahonen CL, Wasiuk A, Fuse S (2008). Enhanced efficacy and reduced toxicity of multifactorial adjuvants compared with unitary adjuvants as cancer vaccines. *Blood*.

[182] Takeda K, Kojima Y, Uno T (2010). Combination therapy of established tumors by antibodies targeting immune activating and suppressing molecules. *Journal of Immunology*.

[183] Dudley ME, Wunderlich JR, Yang JC (2005). Adoptive cell transfer therapy following non-myeloablative but lymphodepleting chemotherapy for the treatment of patients with refractory metastatic melanoma. *Journal of Clinical Oncology*.

[184] Bellone G, Novarino A, Vizio B (2009). Impact of surgery and chemotherapy on cellular immunity in pancreatic carcinoma patients in view of an integration of standard cancer treatment with immunotherapy. *International Journal of Oncology*.

[185] Lake RA, Robinson BWS (2005). Immunotherapy and chemotherapy—a practical partnership. *Nature Reviews Cancer*.

[186] Morse MA, Hobeika AC, Osada T (2008). Depletion of human regulatory T cells specifically enhances antigen-specific immune responses to cancer vaccines. *Blood*.

